# Improving the Reaction Kinetics by Annealing MoS_2_/PVP Nanoflowers for Sodium-Ion Storage

**DOI:** 10.3390/molecules28072948

**Published:** 2023-03-25

**Authors:** Yuan Li, Lingxing Zan, Jingbo Chen

**Affiliations:** 1 School of Materials Science and Engineering, Zhengzhou University, Zhengzhou 450001, China; 2 School of Mechanical Engineering, Henan Institute of Technology, Xinxiang 453000, China; 3 Key Laboratory of Chemical Reaction Engineering of Shaanxi Province, College of Chemistry & Chemical Engineering, Yan’an University, Yan’an 716000, China

**Keywords:** MoS_2_, hollow nanoflower structure, superior conductivity, anode materials, sodium-ion batteries

## Abstract

Under the ever-growing demand for electrochemical energy storage devices, developing anode materials with low cost and high performance is crucial. This study established a multiscale design of MoS_2_/carbon composites with a hollow nanoflower structure (MoS_2_/C NFs) for use in sodium-ion batteries as anode materials. The NF structure consists of several MoS_2_ nanosheets embedded with carbon layers, considerably increasing the interlayer distance. Compared with pristine MoS_2_ crystals, the carbon matrix and hollow-hierarchical structure of MoS_2_/C exhibit higher electronic conductivity and optimized thermodynamic/kinetic potential for the migration of sodium ions. Hence, the synthesized MoS_2_/C NFs exhibited an excellent capacity of 1300 mA h g^−1^ after 50 cycles at a current density of 0.1 A g^−1^ and 630 mA h g^−1^ at 2 A g^−1^ and high-capacity retention at large charge/discharge current density (80% after 600 cycles 2 A g^−1^). The suggested approach can be adopted to optimize layered materials by embedding layered carbon matrixes. Such optimized materials can be used as electrodes in sodium-ion batteries, among other electrochemical applications.

## 1. Introduction

Developing efficient and cost-effective materials is necessary to improve manufacturing processes for affordable electrochemical power storage devices. Such materials can be further used to improve the performance of power storage devices widely used in compact electronics, electrical motor vehicles, and other applications [1,2,3]. The most promising power storage devices developed recently are sodium-ion batteries (SIBs); SIBs have been successfully commercialized and are used extensively [4]. In a typical SIB, electrical energy is stored as chemical energy derived from the reversible intercalation and deintercalation of sodium into and out of anode materials (e.g., graphite) [5,6,7]. The use of recently developed effective anode materials has increased the energy concentration of SIBs [8,9,10].

Owing to its good chemical stability, electronic properties, and high theoretical capacity, two-dimensional molybdenum disulfide (MoS_2_) has received comprehensive focus as a potential substance for use in sodium-ion storage [11,12,13,14]. However, the applicability of MoS_2_ is limited by its weak intrinsic conductivity and considerable volume effect during the process of discharge/charge, similar to graphite [15]. The spacing between adjacent MoS_2_ layers (0.63 nm) is approximately twice as large as that in graphite (0.34 nm). Thus, the intercalation of sodium into MoS_2_ is considerably facile and does not require any significant volume expansion, indicating its potential for use in SIBs [16]. Hence, it can be considered an excellent electrode material for SIBs. Nevertheless, the performance of bulk MoS_2_ as an anode material in SIBs is unsatisfactory because of quick capacity fading owing to its weak electrical/ionic conductivity and structural destruction during sodiation/desodiation [11,15,17].

Several studies have proposed strategies to improve the electrochemical performance of MoS_2_, for example, modifying the structure of pristine MoS_2_, improving structural stability and ion diffusion kinetics by developing a three-dimensional porous structure, enhancing surface defects or interlayer spacing for lowering ion diffusion resistance, and phase engineering to enhance electrical conductivity [18,19]. Furthermore, various modification approaches have been adopted for improving the electrical conductivity of MoS_2_ by fabricating composites such as hybrid graphene/MoS_2_ [20] and polyaniline/MoS_2_ electrode materials [21]. These strategies have been effective in enhancing the process of charge/discharge. Studies have demonstrated the excellent electrochemical performance of hybrid nanocomposites [22,23]. For example, Deng et al. investigated the applicability of a graphene/MoS_2_ mesoporous foam as the anode material in SIBs; the resultant device had a high discharge capacity of 488 mA h g^−1^ at a current density of 100 mA g^−1^ after 100 cycles and a power of 200 mA h g^−1^ at 1000 mA g^−1^ after 1000 cycles [19]. Zheng et al. developed an integrated carbon paper/SiO_2_ template with a mesoporous three-dimensional foam structure that achieved stable performance (at 2000 mA g^−1^) over 1000 cycles with a capacity of 225 mA h g^−1^ [18]. Based on the relationship between structure and function, recent research has demonstrated that a hollow hierarchical structure can help achieve improved electrochemical performance.

Motivated by the aforementioned research, this study established an easy-to-use solvothermal technique for the fabrication of hollow MoS_2_/carbon nanoflowers (NFs) utilizing polyvinylpyrrolidone (PVP) as a carbon precursor and disodium molybdate and thiourea (CH_4_N_2_S) as sources of Mo and S, respectively. By annealing the MoS_2_/PVP NFs in an Ar atmosphere at 600 °C, hollow hierarchical MoS_2_/C NFs with good reversible capability (1300 mA h g^−1^ at 0.1 A g^−1^), strong cycling stability (516 mA h g^−1^ after 600 cycles at 2 A g^−1^), and potent capability (630 mA h g^−1^ at 2 A g^−1^) were generated. In the resulting hollow hierarchical MoS_2_/C NFs, the hollow architecture facilitated a large electrode–electrolyte interfacial area. For rapid sodium-ion diffusion and the expansion of volume during the process of charge/discharge, the hierarchical structures provided sufficiently large voids. Moreover, the conductivity and structural stability of the electrode material were increased by carbon layer doping.

## 2. Results and Discussion

**Scheme 1:**
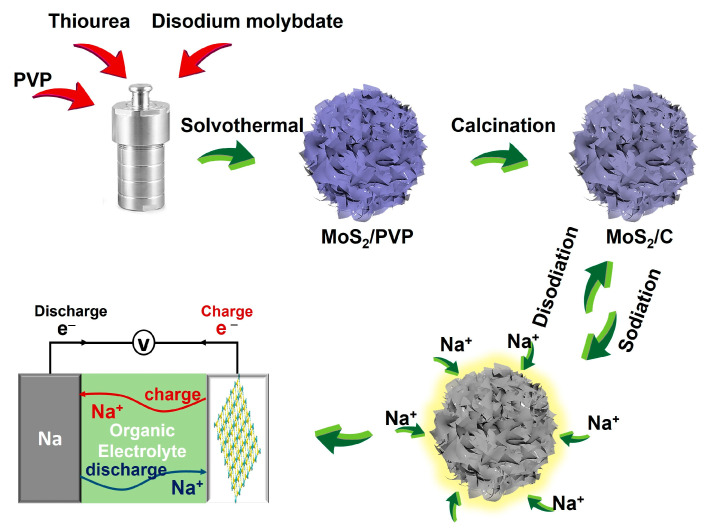
Schematic illustration of the fabrication of hollow MoS_2_/C nanoflowers from diatomite as SIB anodes.

Scheme 1 illustrates the method adopted to fabricate the hollow hierarchical MoS_2_/C NFs. PVP and thiourea were first dispersed in an organic solvent containing dimethylformamide (DMF) and hydrazine hydrate (N_2_H_4_·H_2_O) to obtain a homogeneous solution through ultrasonication. During the chemical reaction at 200 °C, the disodium molybdate and thiourea were employed as sources of Mo and S, respectively. Next, nanosheets consisting of several layers of MoS_2_ nanosheets and PVP were assembled into hollow hierarchical NFs during the hydrothermal process. As reported in the literature, the van der Waals interface between the ultrathin MoS_2_ nanosheets primarily enables the development of hollow hierarchical structures. Subsequently, the hollow MoS_2_/PVP-precursor NFs were annealed in an Ar atmosphere for 2 h at 500 °C, resulting in a carbon layer in or on the MoS_2_ layer and, thus, excellent electrochemical performance.

**Figure 1:**
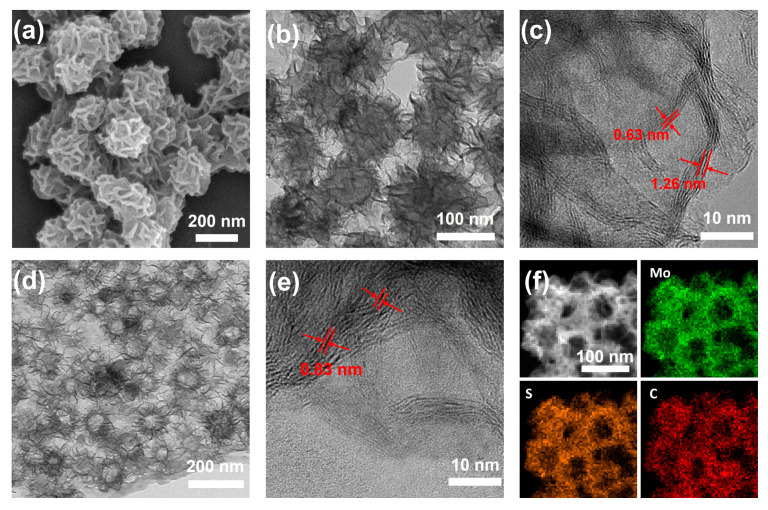
Morphological structure characterizations. (**a**) SEM, (**b**) TEM, (**c**) HRTEM images of MoS_2_. (**d**) TEM, (**e**) HRTEM, and (**f**) mapping images of hollow MoS_2_/C nanoflowers.

**Figure 2:**
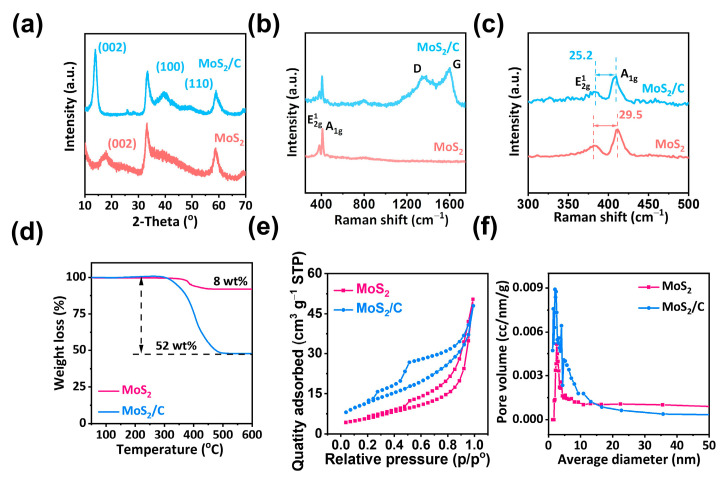
Structural characterization. (**a**) XRD patterns of MoS_2_/C and MoS_2_. (**b**,**c**) Raman patterns of MoS_2_/C and MoS_2_. (**d**) TG patterns of MoS_2_/C and MoS_2_. (**e**) N_2_ adsorption–desorption isotherms and (**f**) pore-size distribution curves of the MoS_2_/C and MoS_2_, respectively.

The images of the hollow MoS_2_/C NFs and MoS_2_ obtained through transmission electron microscopy (TEM; Figure 1a) and standard scanning electron microscopy (SEM; Figure 1b) are depicted in Figure 1. The MoS_2_ layer exhibited a uniform hollow framework. The interlayer distance was estimated to be 0.63 nm (see Figure 1c); this value was consistent with the X-ray diffraction (XRD) results along the (002) plane of the MoS_2_ layer (Figure 2a) [24]. High-angle annular dark field and TEM-STEM images indicated that the MoS_2_/C NFs retained their hollow NF structure despite being doped with carbon. The TEM images in Figure 1d,e indicated that the MoS_2_ NFs had a large interlayer spacing (0.85 nm) and a large number of active sites, which could serve as sites for sodium-ion sodiation/desodiation. Energy-dispersive X-ray spectroscopy (Figure 1f and Figure S1) analysis revealed that the carbon layers on the hollow MoS_2_ NFs were uniformly doped. Furthermore, the XRD patterns indicated the presence of thin carbon layers outside the hollow MoS_2_/C NFs, with no visible C (002) peaks except for the diffraction peaks corresponding to MoS_2_ after carbon layer doping (see Figure 2a). In the Raman spectra of the hollow MoS_2_/C NFs, an additional characteristic G peak (1582 cm^−1^) indicated the presence of graphite in addition to the A_1g_ (408 cm^−1^) and E1_2g_ (383 cm^−1^) forms of MoS_2_ (see Figure 2b) [25]. The D band (1370 cm^−1^) in the Raman spectra indicated defects in the carbon layers, consistent with the TEM results (Figure 1e). In addition, a large interlayer spacing was revealed by local Raman amplification (Figure 2c) [26]. Thermogravimetric analysis (TGA) revealed that the hollow MoS_2_/C NFs had a carbon content of 44 wt% (Figure 2d), which increased the specific surface area. The hollow NF structures and the ultrasmall dimensions of the nanosheets endow both of them with a relatively large surface area of 22.7 m^2^ g^−1^ and 43.1 m^2^ g^−1^, respectively (Figure 2e), as revealed by Brunauer–Emmett–Teller (BET) analysis. Moreover, the BET surface area curve for the hollow MoS_2_/C NFs was a Type IV isotherm (Figure 2e) with a clear H_3_ hysteresis loop, demonstrating the presence of adequate mesopores with diameters of approximately 6.9 nm inside the complex material (Figure 2f).

**Figure 3:**
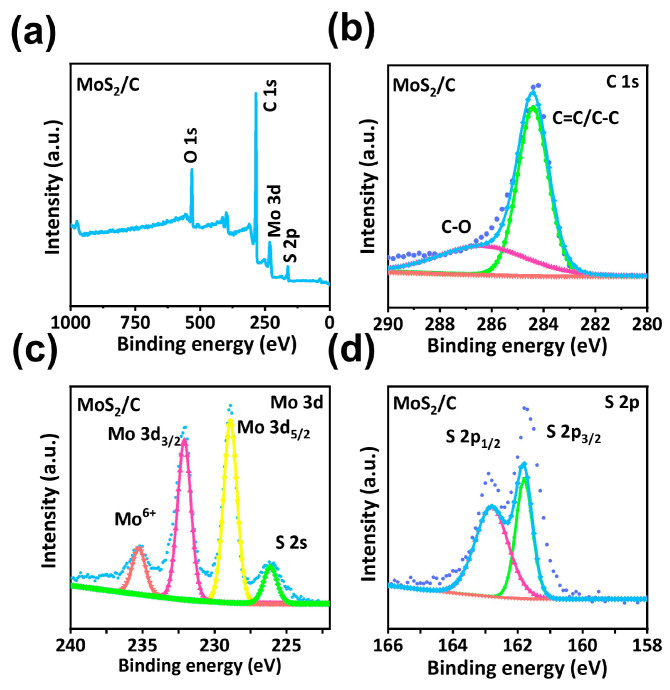
Structural characterization. (**a**) XPS survey scan of hollow MoS_2_/C nanoflowers. High-resolution (**b**) C 1s, (**c**) Mo 3d and (**d**) S 2p spectra of MoS_2_/C, respectively.

The chemical composition and surface electronic states of the hollow MoS_2_/C NFs were determined by employing X-ray photoelectron spectroscopy (XPS). Figure 3a depicts the Mo 3d, S 2p, O 1s, and C 1s spectra of the structures. The peaks at 284.3 and 286.5 eV in the C 1s spectra (Figure 3b) can be attributed to C–O and C–C/C=C bonds [27]. In the high-resolution Mo 3d spectra (Figure 3c), the peaks at 226.3, 228.8, 232.1, and 235.2 eV correspond to S 2s, Mo 3d^5/2^, Mo 3d^3/2^, and Mo^6+^, respectively. The peak at 235.2 eV can be attributed to Mo^6+^ 3d^3/2^, which is generated by the surface oxidation of Mo^4+^ in an air atmosphere [28]. The two peaks at 161.7 and 162.8 eV in the S 2p spectra can be attributed to S 2p^3/2^ and S 2p^1/2^, respectively (Figure 3d) [29].

**Figure 4:**
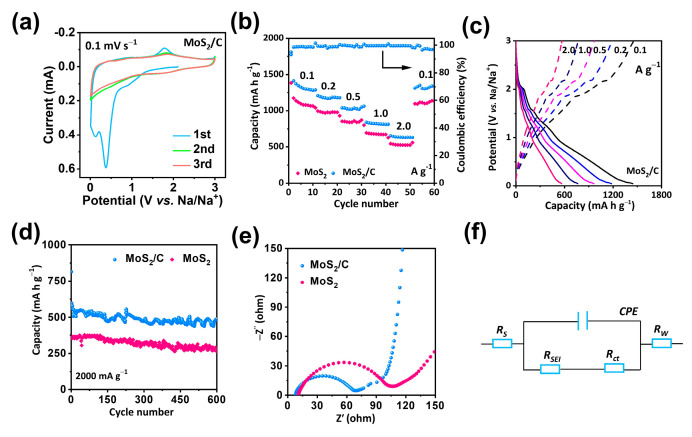
Sodium-ion storage performance. (**a**) CV curves of MoS_2_/C at scan rate of 0.1 mV s^−1^. (**b**) Rate performances at various current densities. (**c**) The GCD curves of MoS_2_/C at various current densities. (**d**) Long-term cycling stability at 2.0 A g^−1^. (**e**) EIS curves of MoS_2_/C and MoS_2_. (**f**) Equivalent circuit model corresponding to the Nyquist plots of the MoS_2_/C.

In a voltage range of 0.05–3.0 V vs. Na/Na^+^, the electrochemical storage capability of the hollow composite MoS_2_/C anode was measured and subjected to a comparison with that of a pure MoS_2_ anode. First, cyclic voltammograms (CV) were recorded for the initial three cycles of the hollow MoS_2_/C NFs at a scan rate of 0.1 mV s^−1^ (Figure 4a). A reduction peak was found at 0.42 V during the first discharge cycle, associated with the intercalation of the sodium ion into MoS_2_ and the generation of a solid–electrolyte interface (SEI) film. A prominent oxidation peak was observed within the range of 1.5–2.0 V, corresponding to the deintercalation of Na_x_MoS_2_ to MoS_2_ and sodium metal produced during the first charge cycle [30]. The oxidation and reduction peaks were roughly constant from the third cycle onwards, indicating the excellent reversibility and cycling stability of the hollow MoS_2_/C NF electrode throughout the sodiation/desodiation processes. The electrochemical performance of hollow MoS_2_/C NFs was further examined by analyzing the rate performance and cycling stability. As illustrated in Figure 4b,c, the hollow MoS_2_/C NFs exhibited the highest rate capacities of 1321, 1171, 1036, 819, and 632 mA h g^−1^ at current densities of 0.1, 0.2, 0.5, 1.0, and 2.0 A g^−1^, respectively. Furthermore, the discharge capacity recovered to 1300 mA h g^−1^ when the current density reduced from 2.0 to 0.1 A g^−1^. These results indicated the outstanding reaction kinetics of the hollow MoS_2_/C NFs, attributable to the rapid electron transport and sodium-ion diffusion induced by the carbon and MoS_2_ layer, thereby resulting in the high utilization of MoS_2_ NFs and a high specific capacity. Additionally, long-term cycling performance at high current density was achieved. As depicted in Figure 4d, the hollow MoS_2_/C NFs retained a high reversible capacity of 438 mA h g^−1^ even after 600 cycles at 2.0 A g^−1^, with a slow decay rate of 0.034% per cycle. Without the carbon layer structure, the MoS_2_ NFs exhibited the lowest specific capacity and rate stability. 

**Figure 5:**
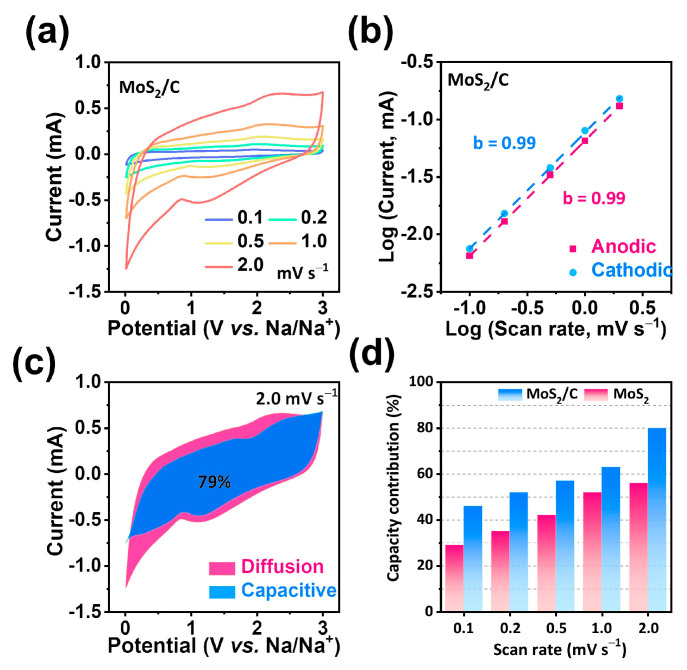
Electrochemical kinetic behaviors. (**a**) CV curves of hollow MoS_2_/C nanoflowers at various current densities. (**b**) Log(i)-log(*v*) plots at different cathodic/anodic peaks. (**c**) Normalized contribution ratios of capacitive capacities of MoS_2_/C at a scan rate of 2.0 mV s^−1^. (**d**) Normalized contribution ratios of capacitive capacities MoS_2_/C and MoS_2_ at different scan rates.

At an electrolyte/electrode interface, EIS technology is an efficient technique to determine the kinetics of charge transfer [31,32,33]. In the Nyquist plots shown in Figure 4e, a single depressed semicircle in the high-frequency region indicated the charge transfer resistance (Rct), and the sodium-ions diffusion ability, also known as the Warburg impedance (Zw), was indicated by the inclined line in the low-frequency region [34,35,36]. The inset of Figure 4f displays the equivalent circuit that corresponds to the Nyquist impedance plots, along with definitions for each parameter. In accordance with the equivalent circuit model, the hollow MoS_2_/C NFs exhibited a lower Rct value (60 Ω) than that of MoS_2_ (93 Ω), indicating that the hollow MoS_2_/C NFs can remarkably enhance interfacial charge separation and transfer between adjacent layers when sodium-ions are repeatedly inserted/extracted. The present study also investigated the sodium-ion storage kinetics of hollow MoS_2_/C NFs through CV measurements, as illustrated in Figure 5a. data on the scan rates between 0.1 and 2.0 mV s^−1^ were employed to obtain CV curves. As illustrated in Figure 5b, the b values calculated using the linear relationship between log (current) and log (scan rate) during anodic and cathodic scans were 0.99 and 0.99, respectively, indicating that the process was capacitance-controlled [37]. As shown in Figure 5c and Figure S4, the contribution of capacitive charge was 79% at a scan rate of 2.0 mV s^−1^. Therefore, compared with MoS_2_ (Figure S3), hollow MoS_2_/C NFs exhibited larger capacitive components at all scan rates, thus indicating greater efficient sodium-ion adsorption and quicker reaction kinetics [38].

The electrode was recycled after 600 cycles at 2.0 A g^−1^ to determine the factors contributing to the high performance of hollow MoS_2_/C NFs. TEM and XPS were used to analyze the morphology and nanostructure of the regenerated hollow MoS_2_/C NFs. The results indicated that the morphology and structure of the NFs remained unchanged in the presence of long-term cycling, indicating the electrode’s high durability owing to the carbon layers. Although the macroporous nanostructure of the hollow MoS_2_/C NFs partially collapsed, the porosity of the structure was still evident in the TEM images displayed in Figures S5 and S6. The HRTEM image of MoS_2_ shown in Figure S6b indicated the presence of the layered structure even after cyclic use. However, the interlayer spacing of the hollow MoS_2_/C NFs exhibited a substantial increase (up to 1.03 nm in contrast with a layer spacing of 0.83 nm in the new electrode), likely because of long-term use in sodiation/desodiation, which helps minimize mass transfer resistance and favors long-term cyclic stability. As indicated by the XPS results shown in Figure S7, the hollow MoS_2_/C NF electrodes retained their good electrical structure even after prolonged cyclic use.

## 3. Materials and Methods

### 3.1. Chemicals

From Sinopharm Chemical Reagent Co., Ltd. (Shanghai, China), PVP, DMF, hydrazine hydrate, sodium molybdate dehydrate (Na_2_MoO_4_), and thiourea were procured. None of the chemicals used were further purified since they were all analytical-grade chemicals.

### 3.2. Synthesis of Hollow MoS_2_/Carbon NFs

A solution comprising 35 mL of DMF and 0.1 mL of hydrazine hydrate was used to thoroughly dissolve 0.01 g PVP and 0.06 g thiourea in a typical synthesis. Then, the addition of 0.02 g of sodium molybdate dehydrate was carried out. Following 30 min of stirring, the solution was heated under static circumstances in a Teflon-lined autoclave for 24 h at 200 °C. The recovery of a solid product was achieved through centrifugation in water and ethanol thrice, and the product was vacuum-dried overnight at 60 °C. Precursor samples were prepared by pyrolyzing the solid in an Ar atmosphere in a tube furnace at a heating rate of 2 °C min^−1^ up to 250 °C, dwelling for 2 h, reheating at a rate of 5 °C min^−1^ up to 500 °C, and dwelling again for 2 h.

### 3.3. Synthesis of MoS_2_ NFs

To facilitate the comparison, the aforementioned process was also applied to prepare the MoS_2_ sample, but PVP was not added.

### 3.4. Materials Characterization

An FEI Quanta 200F was employed for conducting SEM. High-resolution transmission microscopy (HRTEM) was performed using an FEI TECNAI G2 F30. PE Diamond TG/DTA was employed for high-resolution TGA. KANGTA Quadrasorb evo was used for BET. XRD was performed using a Rigaku D/Max 2500/PC. LabRAM HR 800 Raman spectrometer was employed for Raman spectroscopy. KRATOS Axis Ultra DLD was employed for XPS.

### 3.5. Electrochemical Measurements

CR2032 coin cells were employed for electrochemical experiments. The working electrodes of the cells comprised an active material, carbon black, and a polymer binder (polyvinylidene fluoride) mixed at a weight ratio of 7:2:1. The electrodes were constructed by coating this mixture onto Cu foil and drying it under vacuum for 24 h at 60 °C. The electroactive materials had a mass loading of 0.6–0.8 mg cm^−2^. As counter electrodes and reference electrodes, metallic Na pieces were utilized. As a separator, a glass fiber membrane (Whatman/F) was employed. The electrolyte was constituted by a 1 M solution of NaClO_4_ in a 1:1 vol% mixture of ethylene carbonate/diethyl carbonate with 5 wt% fluoroethylene carbonate. A NEWARE battery testing system was employed for galvanostatic discharge/charge measurements within a voltage range of 0.05–3.0 V (vs. Na/Na^+^). The electrolyte and separator were identical to those used in the half cell, and the voltage range was between 0.05 and 3.0 V. An electrochemical workstation (CHI 760E) was employed for CV and electrochemical impedance spectroscopy (EIS) tests. By applying a 5 mV amplitude signal within the frequency range 50–100 kHz, electrodes’ EIS spectra were obtained.

## 4. Conclusions

We designed and synthesized a unique hollow MoS_2_/C NFs with large interlayer spacing for SIB applications. The large interlayer spacing and the carbon layer in the as-prepared composite material provide ion adsorption sites and diffusion channels for the transportation of ions. Furthermore, the conductivity of MoS_2_ was improved because of favorable interactions between the carbon and the MoS_2_ layers in the prepared material. Therefore, the designed MoS_2_/carbon NF composites can exhibit outstanding long-term cycling performance with a capacity of 438 mA h g^−1^ after 600 cycles at 2.0 A g^−1^. The current study offers novel perspectives into the structural design of 2D materials as energy storage materials by highlighting the importance of enhancing the sodium storage performance of MoS_2_ electrodes by increasing interlayer spacing and incorporating carbon layers.

## Data Availability

The data that support the findings of this study are available from the corresponding author upon reasonable request.

## Supplementary Materials

The following supporting information can be downloaded at: https://www.mdpi.com/article/10.3390/molecules28072948/s1, Figure S1: EDS analysis of MoS_2_/C; Figure S2: XPS survey scan of MoS_2_. High-resolution (b) Mo 3d and (c) S 2p spectra of MoS_2_, respectively; Figure S3: (a) CV curves at various scan rates of MoS_2_. (b–f) Capacitive-controlled and diffusion-controlled contributions at different scan rates of MoS_2_; Figure S4: (a–d) Capacitive-controlled and diffusion-controlled contributions at different scan rates of MoS_2_/C; Figure S5: Morphology and nanostructure characterizations of MoS_2_ electrode after 600 long-term cycles under the current density of 2.0 A g^−1^; Figure S6: Morphology and nanostructure characterizations of MoS_2_/C electrode after 600 long-term cycles under the current density of 2.0 A g^−1^; Figure S7: (a) XPS full spectra and high-resolution (b) C 1s, (c) Mo 3d, and (d) M S 2p spectra of MoS_2_/C after long-term cycles; Table S1: Comparison of the rate and cycle performance between MoS_2_/C NFs with the recently reported MoS_2_-based anode materials. Ref [18,23,25,30,39,40] are cited in the supplementary materials.
