# Supplementary material for: Improving the Reaction Kinetics by Annealing MoS2/PVP Nanoflowers for Sodium-Ion Storage
Source: Molecules. 2023 Mar 25;28(7):2948. doi: 10.3390/molecules28072948 (PMC10095949; doi:10.3390/molecules28072948)
Supplement: Supplementary file 1 [file molecules-28-02948-s001.zip › molecules-2246567-supplementary.pdf]

Supporting Information

Improving the Reaction Kinetics by Annealing MoS_2/PVP Nanoflowers for Sodium-Ion Storage

Yuan Li [1,2], Lingxing Zan [3,*] and Jingbo Chen [1,*]

[1] School of Materials Science and Engineering, Zhengzhou University, Zhengzhou 450001, China
[2] School of Mechanical Engineering, Henan Institute of Technology, Xinxiang 453000, China
[3] Key Laboratory of Chemical Reaction Engineering of Shaanxi Province; College of Chemistry & Chemical Engineering, Yan'an University, Yan'an 716000, China
Correspondence: zanlingxing@yau.edu.cn (L.Z.); chenjb@zzu.edu.cn (J.C.)

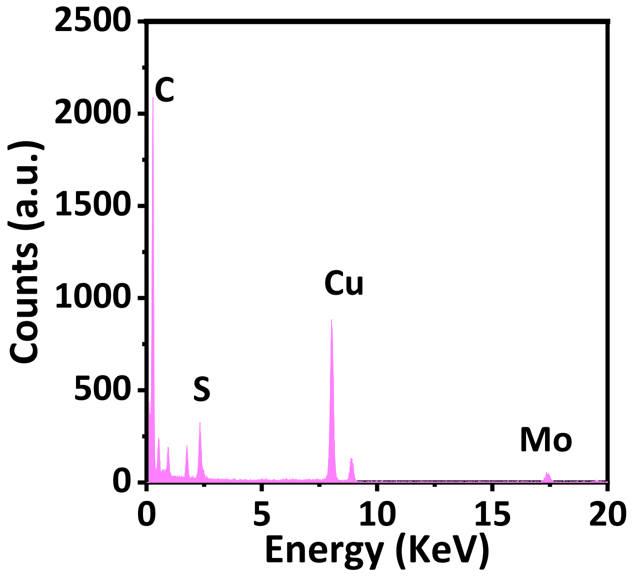

Figure S1. EDS analysis of MoS_2/C.

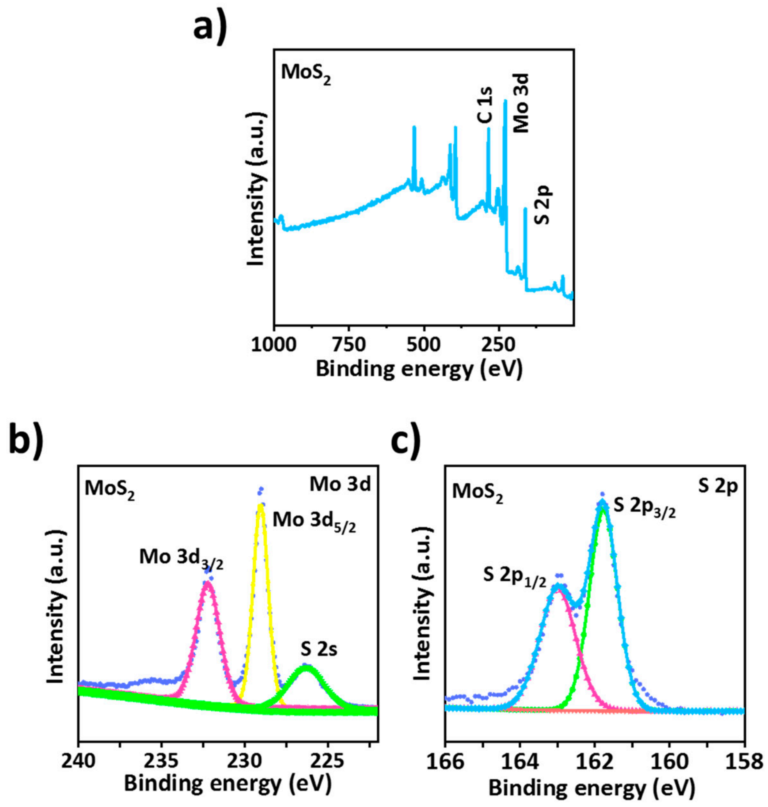

Figure S2. a) XPS survey scan of MoS_2. High-resolution b) Mo 3d and c) S 2p spectra of MoS_2, respectively.

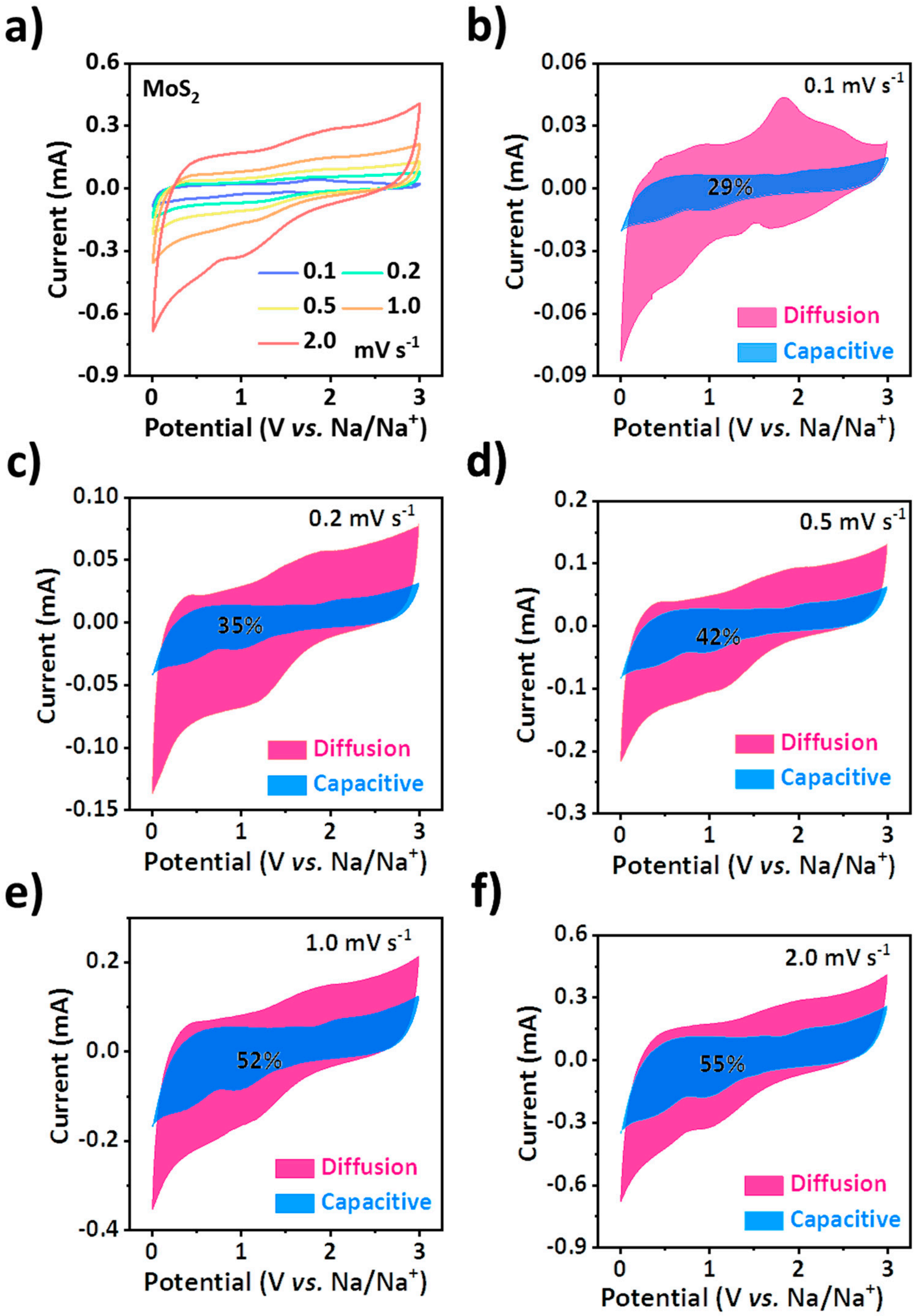

Figure S3. a) CV curves at various scan rates of MoS_2. b-f) Capacitive-controlled and diffusion-controlled contributions at different scan rates of MoS_2.

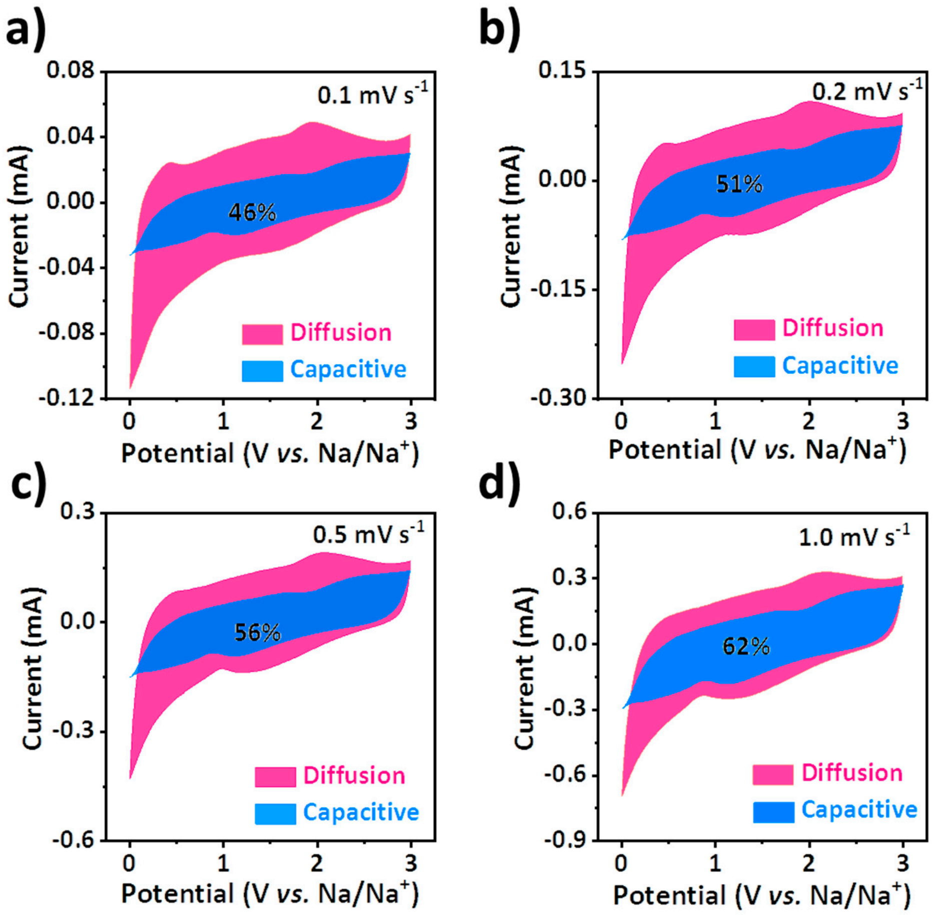

Figure S4. a-d) Capacitive-controlled and diffusion-controlled contributions at different scan rates of MoS_2/C.

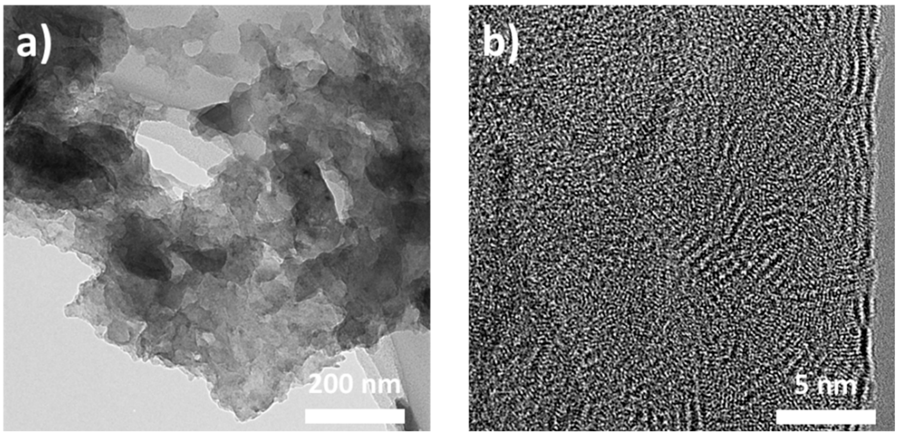

Figure S5. Morphology and nanostructure characterizations of MoS_2 electrode after 600 long-term cycles under the current density of 2.0 A g^{-1}.

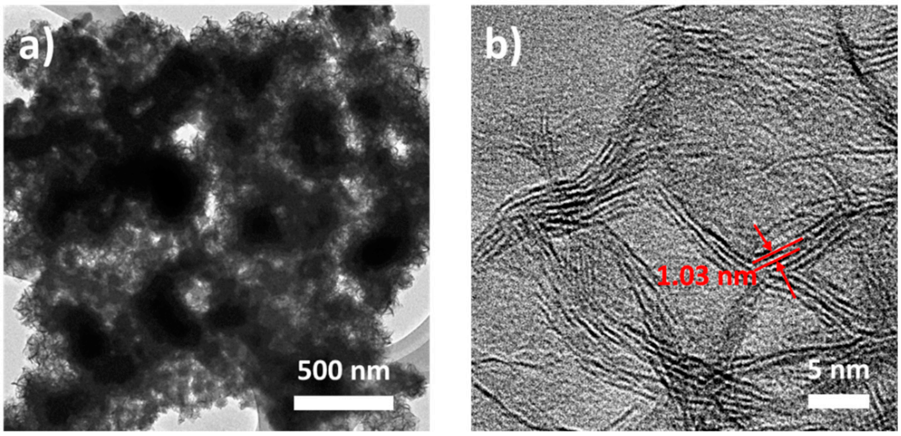

Figure S6. Morphology and nanostructure characterizations of MoS_2/C electrode after 600 long-term cycles under the current density of 2.0 A g^{-1}.

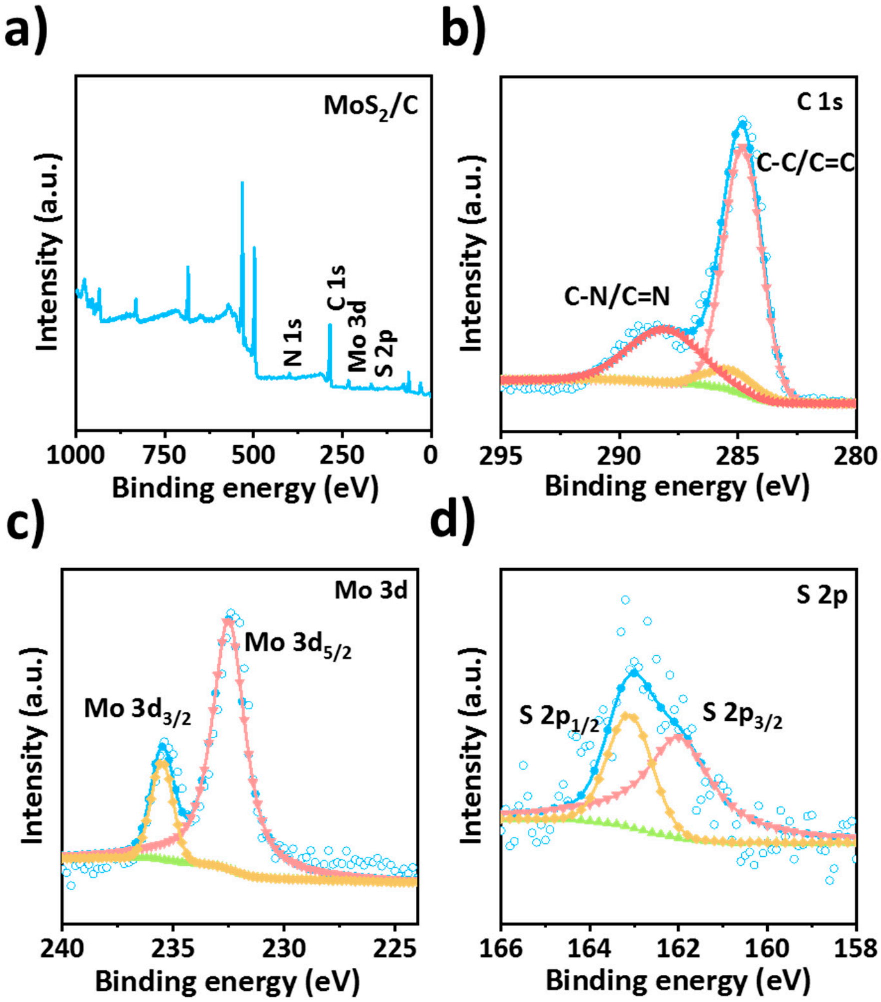

Figure S7. a) XPS full spectra and high-resolution b) C 1s, c) Mo 3d, and d) M S 2p spectra of MoS_2/C after long-term cycles.

Table S1. Comparison of the rate and cycle performance between MoS_2/C NFs with the recently reported MoS_2-based anode materials.

	Sample	Voltage	Rate	Cycle	Reference
	**MoS_2/C NFs**	0.01-3.0 V	1321, 1171, 1036, 819, and 632 mA h g^{-1} at 0.1, 0.2, 0.5, 1.0, and 2.0 A g^{-1}	438 mA h g^{-1} at 2.0 A g^{-1} after 600 cycles	***This work***
1	MoS_2/C	0.01-3.0 V	465, 427, 387, 352, and 313 mA h g^{-1} at 0.1, 0.2, 0.5, 1.0, and 2.0 A g^{-1}	53 mA h g^{-1} at 2.0 A g^{-1} after 2000 cycles	[1]
2	3D MoS_2/CP	0.4-3.0 V	490, 438, 377, 329, 274, and 225 mA h g^{-1} at 0.05, 0.1, 0.3, 0.5, 1.0, and 2.0 A g^{-1}	225 mA h g^{-1} at 2.0 A g^{-1} after 1000 cycles	[2]
3	Cu2S@C@MoS2	0.1-3.0 V	430, 410, 386, 368, 359, 337, and 316 mA h g^{-1} at 0.05, 0.1, 0.2, 0.3, 0.5, 1, and 2 A g^{-1}	309 mA h g^{-1} at 0.3 A g^{-1} after 200 cycles	[3]
4	MoS_2@SnO_2	0.01-3.0 V	525, 444, 287, 195, and 114 mA h g^{-1} at 0.1, 0.5, 1.0, 2.0, and 5.0 A g^{-1}	262 mA h g^{-1} at 2.0 A g^{-1} after 500 cycles	[4]
5	MoS_2/CNT	0.01-3.0 V	460, 346, 310, 230, and 146 mA h g^{-1} at 1.0, 2.0, 5.0, 10, and 20.0 A g^{-1}	280 mA h g^{-1} at 2.0 A g^{-1} after 500 cycles	[5]
6	MoS_2/C	0.0-3.0 V	530, 500, 370, 250, and 230 mA h g^{-1} at 0.1, 0.2, 0.5, 1.0, 2.0 A g^{-1}	256 mA h g^{-1} at 1.0 A g^{-1} after 200 cycles	[6]

[1] Xia, H.; Yuan, P.; Zan, L.; Qu, G.; Dong, H.; Wei, Y.; Yu, Y.; Wei, Z.; Yan, W.; Hu, J.S.; et al. Evolution of Stabilized 1T-MoS2 by Atomic-Interface Engineering of 2H-MoS2/Fe-Nx towards Enhanced Sodium Ion Storage. *Angew. Chem. Int. Ed.* **2023**, *62*, e202218282.

[2] Zheng, F.; Wei, Z.; Xia, H.; Tu, Y.; Meng, X.; Zhu, K.; Zhao, J.; Zhu, Y.; Zhang, J.; Yang, Y.; et al. 3D MoS2 foam integrated with carbon paper as binder-free anode for high performance sodium-ion batteries. *J. Energy Chem.* **2022**, *65*, 26–33.

[3] Fang, Y.; Luan, D.; Chen, Y.; Gao, S.; Lou, X.W. Rationally Designed Three-Layered Cu2S@Carbon@MoS2 Hierarchical Nanoboxes for Efficient Sodium Storage. *Angew. Chem.-Int. Ed.* **2020**, *59*, 7178–7183.

[4] Gui, D.; Wei, Z.; Chen, J.; Yan, L.; Li, J.; Zhang P.; Zhao C. Boosting the sodium storage of the 1T/2H MoS 2@ SnO 2 heter-ostructure via a fast surface redox reaction. *J. Mater. Chem. A.* **2021**, *9*, 463–471.

[5] Han, W.; Xia, Y.; Yang, D.; Dong, A. Exfoliation of large-flake, few-layer MoS 2 nanosheets mediated by carbon nanotubes. *Chem. Communications*. **2021**, *57*, 4400–4403

[6] Wang, Y.; Yang, Y.; Zhang, D.; Wang, Y.; Luo, X.; Liu, X.; Kim, J.K.; Luo, Y. Inter-overlapped MoS2/C composites with large-interlayer-spacing for high-performance sodium-ion batteries. *Nanoscale Horiz.* **2020**, *5*, 1127–1135
